# Correlation between levels for stress with level of empathy in undergraduate medical students

**DOI:** 10.12669/pjms.39.5.7211

**Published:** 2023

**Authors:** Shumaila Rafi, Marie Andrades, Rahat Naz, Asad Jiskani

**Affiliations:** 1 Shumaila Rafi, FCPS, MHPE Associate Professor Medicine Al- Tibri Medical College Karachi (Isra University Karachi Campus), Pakistan; 2 Marie Andrades, FCPS, MHPE Head of Institute of Family Medicine Jinnah Sindh Medical University, Karachi, Pakistan; 3 Rahat Naz, MHPE, MBA(HHCM) Director Affiliated Colleges Jinnah Sindh Medical University, Karachi, Pakistan; 4 Asad Jiskani, MPH, MHPE Director Medical Education Al- Tibri Medical College Karachi (Isra University Karachi Campus), Pakistan

**Keywords:** Empathy, Stress, Jefferson Scale of Empathy, Perceived Stress Scale, Medical students

## Abstract

**Objective:**

To determine the correlation between levels of stress with level of empathy in all five years of undergraduate medical students of a private medical college in Pakistan.

**Methods:**

This descriptive correlation study was conducted at Al Tibri Medical College, Karachi from 15^th^ June to 14^th^ November 2021. Of the 500 students in the medical school, 408 participants filled out the questionnaires through online Google Forms. The student’s version of the Jefferson Scale of Empathy (JSE-S) estimated the self-reported student’s empathy levels. At the same time, Perceived Stress Scale (PSS) was utilized to assess the student’s levels of stress. Data was analyzed using SPSS version 22.0 and correlation between empathy and perceived stress was calculated by Pearson’s coefficient. A p-value <.05 was considered statistically significant.

**Results:**

Out of 408 participants, there were 217(53.2%) males, and 191(46.8%) females. The overall mean JSE-S score was 94.60±11.85, and the mean PSS score was 20.20 ±5.70. Empathy scores improved over the basic sciences years and then significantly decreased in the clinical years of medical college with a significant p-value of .019. The highest stress was present in third year medical students with a p-value of <.001. No statistically significant difference was present between empathy and stress levels (r = 0.04, p = .40).

**Conclusions:**

The study showed no statistically significant correlation between empathy and stress. Future research is needed to investigate other main factors for the decline in empathy among medical students.

## INTRODUCTION

Empathy is fundamental to providing the best possible care for patients.^1^ The central skills suggested by the Accreditation Council for Graduate Medical Education (ACGME) incorporate medical student professionalism^2^, with a distinctive characteristic of medical professionalism being empathy.^3^ Empathy is an important determinant of the healthcare quality, and is identified as the quintessential personal value of humanistic physicians.^4^ This creates belief and confidence in the rapport of doctor and patient fulfillment and the patient’s capacity to adapt to stress.^5^

World Health Organization stated that the epidemic of the 21st century is stress.^6^ At least one form of distress is present in more than 80% of medical students, of which stress, depression, and burnout are the most common.^7^ Stress is higher among medical students in developing countries such as Pakistan, India, Malaysia, and Thailand; the studies also highlighted the task of academics as an imminent source of stress.^8^ Hojat et al. reported that medical students achieved considerably less empathy scores after entering the clinical years of medical college. It was established that there was a widespread drop in empathy in year three of the medical institutions, wherein the focus changed more to patient-targeted activities.^4^,^9^ One study suggested that the consequent worsening may be related to fatigue, stress, and appropriation of problem-based methods in contrast to a humanistic attitude throughout clinical education.^10^ Stress factors in the pre-clinical years are generally notorious with the undergraduate new teaching strategies, academic performance, increased workload, and environmental factors. Clinical years can likewise be stressful with intense academic pressure, lack of role models, lack of time, and ethical dilemmas when managing critical patients.^11^

In Pakistan, more than 90% of medical students reported the presence of stress in their medical training.^12^ Stress can lead to poor educational performance and lower empathy. Medical students are also prone to suicidal ideation due to academic stress.^12^ Therefore, it is crucial to see how medical students manage the stress that affects the decline in empathy throughout the educational process. Globally there is literature available, but we were unable to find any previous study on this topic in Pakistan. Therefore, the study was conducted to correlate stress and empathy among undergraduates. The research aimed to determine the correlation between stress and empathy to build interventions during medical school to combat stress and enhance empathy in students.

## METHODS

This descriptive correlation study was conducted from 15^th^ June to 14^th^ November 2021 at Al Tibri Medical College Karachi**.** Thomas Jefferson University allowed the use of the JSE-S version for undergraduate students. All 500 medical students were requested to participate in the research. It was a universal sampling technique (convenient sampling).

The first year to the last year of undergraduates of MBBS were included in this study. Exclusion criteria included the students who did not gave consent. Data was collected via an online Google Form. The link to the Google Form was distributed to all undergraduate medical students through the WhatsApp group. An Electronic consent form was attached to the Google Form. Before the study, a pilot study of the tools was administered among 25 medical students of five years who were enrolled in another private medical college. The reliability of the pilot study had a Cronbach’s alpha value of 0.74 for the JSE-S scores and 0.79 for the PSS scores.

JSE- S scale consisted of demographic data that included age, gender, specialty plan to choose in the future, and year of medical college. The JSE scale was used in its original form in English. It constituted 20 questionnaires replied on a seven-point Likert scale. The questionnaire comprised both positive and negative items to reduce the bias.^13^ JSE appears to have satisfactory internal consistency, test-retest reliability, and validity.^13^ The higher the score, the upper the level of empathy, with an overall score between 20 to140.^13^

The PSS is a 10-item self-administered tool that assesses the extent of perceived stress in daily activities over the previous month. It has good validity and reliability^14^. It composes six positively and four negatively worded items to be rated on a five-point Likert scale. Scores range from 0 to 40, with greater scores demonstrating higher levels.^14^

### Ethical Approval:

Approval was taken from the Institutional Review Board of Al-Tibri Medical College (IRB# ATMC/IERC/02-2021/01, Dated: June 9, 2021).

Data analysis: Statistical Package for Social Sciences (SPSS) Version 22 was used for data analysis. The Cronbach’s α coefficient was calculated to confirm the reliability of the scores. Frequencies and percentages were obtained for categorical variables. The continuous variables were expressed as mean and standard deviation. An Independent t-test was used to assess difference in empathy level on the basis of gender and analysis of variance (ANOVA) was utilized to analyze differences on the basis of the academic year. The correlation between empathy and stress was assessed by using Pearson’s correlation analysis. The P-value of <.05 was considered statistically significant.

## RESULTS

Of the 500 students in the medical school, 92 (18.4%) students were excluded as they did not provide consent. Of the 408 participants, 217 (53.2%) were males, and 191 (46.8%) were females. The response rate was 408 out of 500. The individual response rate was 70 (17.2%), 83 (20.3%), 90 (22.1%), 80 (19.6%), and 85 (20.8%) year one to year five medical students respectively. All 408 students filled out the questionnaires.

**Fig.1:**
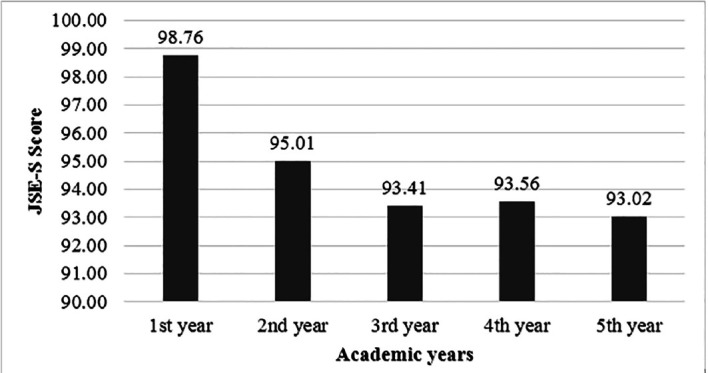
Mean scores of empathy levels of students in academic years (n=408)

**Fig.2:**
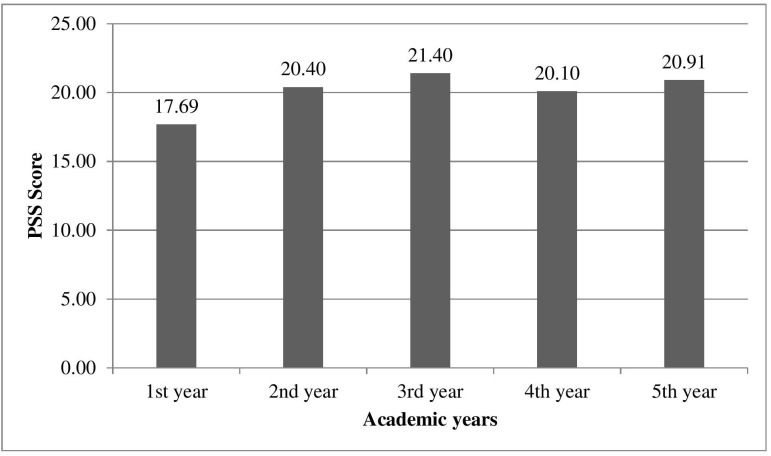
Mean scores of stress levels of students in academic years (n=408)

Majority 234 (57.4%), were younger than 22 years old. The overall mean JSE-S scores were 94.60 ± 11.85. The empathy scores were highest (98.76 ± 11.20) in 1^st^ year and lowest (93.02 ± 11.76) in final year as shown in Fig.1. Independent sample t-test showed that the mean empathy scores and Standard Deviation (SD) were 102.3±12 for females and 98.7±12.1 for males with a p-value of 0.003. The mean PSS scores were 20.20 ± 5.70 and the mean stress scores and SD for females were 20.81±5.62 and 19.59 ±5.87 for males with a p-value of 0.033. Fig. 2 shows the mean scores of stress levels of students in all academic years.

**Fig.3:**
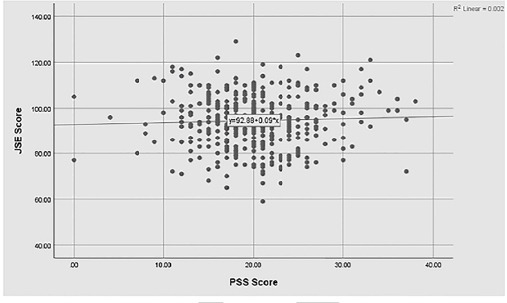
Correlation between JSE-S and PSS in relation with academic years (n=408)

The ANOVA tests showed significant differences in empathy levels among undergraduate medical students, highest in the first year of education. In 3^rd^, 4^th^ and final clinical years, empathy decreased with the lowest in the last year to a significant p-value of 0.019. ANOVA test also showed the stress levels were lowest in year one and peaked in the third year with a significant p-value of 0.001, as shown in Table-I. Fig.3: showed that Pearson’s correlation coefficient indicated no overall correlation between empathy and stress levels (r = 0.04, p = 0.40).

**Table-I:** The empathy and stress levels in diverse groups of academics in undergraduates (n=408)

		N	Mean	Std. Deviation	95% Confidence Interval for Mean		P-value

					Lower Bound	Upper Bound	
JSE score	1st year	70	98.76	11.20	96.09	101.43	.019*
	2nd year	83	95.01	11.06	92.60	97.43	
	3rd year	90	93.41	12.35	90.82	96.00	
	4th year	80	93.56	12.12	90.86	96.26	
	5th year	85	93.02	11.76	90.49	95.56	
	Total	408	94.60	11.85	93.45	95.76	
PSS score	1st year	70	17.69	5.42	16.39	18.98	<.001*
	2nd year	83	20.40	5.03	19.30	21.50	
	3rd year	90	21.40	5.52	20.24	22.56	
	4th year	80	20.10	6.03	18.76	21.44	
	5th year	85	20.91	5.88	19.64	22.17	
	Total	408	20.20	5.70	19.65	20.76	

*. Significant level ≤.05

## DISCUSSION

In this research, the mean empathy rating on the JSE-S scale was found to be higher than other studies reported from Pakistan.^13^,^15^ These results of empathy scores in this study were lower as compared to other Asian countries like Korea (105.8)^7^, and China (102.8).^16^ Medical curricula vary by medical institution, and cultural and social differences in different countries contribute to lower empathy.^17^ In this study, female students were more empathetic than male students. These findings were consistent with different studies in Asia^16^, ^18^, and other Western countries.^19^ Females are more empathetic and caring than men due to social learning and hereditary factors.^20^

The results showed that empathy was higher during the first two years of undergraduate medical education. It could be due to the lesser workload with more leisure time and more enthusiasm in their career. In clinical years, the decrease in empathy can be due to the busy schedules of faculty members who could not be role models for students. This finding was in agreement with Shaheen A et al.^13^ and Chen DC et al.^21^ Dissimilarity in the study of Bangash from Pakistan reported that students scored the same for empathy in their first and final year.^22^ The reason for the comparable levels of empathy in the first and final year was that their institution taught ethics and behavioral sciences in the third year, which improved patients’ communication skills and empathy.^22^ The use of Empathy Quotient Questionnaire scale might also contribute to the differences in the previous study.^22^

The third-year undergraduates were most stressed than other professional years. This contrast may be because standing at the bedside with a large group of people for long periods during the history and examination can cause fatigue and lead to stress, which reduces empathy. This finding supports that of a previous study.^23^ In one study, the highest stress was present in first-year students, which may have been due to an increase in the number of exams and faced challenges as they were exposed to a competitive and ambitious environment.^7^

No correlation was found between levels of empathy and stress. This response may be because students from different cultures interpret and process stressors differently. This finding is in consistent with the previous studies.^11^ The Korean study reported that stress-induced exhaustion and depersonalization led to a drop in empathy.^7^ This difference may also be because the inclusion criteria consisted of undergraduate and postgraduate medical students, and the sample size was large.^7^

The Pakistan Medical Commission (PMC) states that empathy is a key feature for skills acquisition in undergraduate organizations and a characteristic of increasing health professionals in the MBBS program.^24^ Empathy is crucial in clinical settings as it improves the doctor- patient relationship, enhances communication, facilitate holistic care and prevents medical errors.^5^ Therefore, appropriate educational interventions and instructional design in medical curricula are needed to improve the attitudes of our potential doctors and strengthen the healthcare system. In general, simulated patients tended to document a better extent of gratification during meetings with students. Role playing exploring audio or videotaped experiences, and being disclosed to mentors are techniques utilized in interventions designed to augment empathy.^25^ Future research needs to explore other major factors behind the deterioration in empathy among undergraduates and how stress affects empathy among undergraduate medical students. Future research should also be aimed toward emerging multi-institutional studies in Pakistan to understand the relationship between stress levels and empathy as the undergraduate medical year progresses.

### Limitations:

First, the generalizability of the conclusions was limited because of the cross-sectional study of a single institution. The utilization of self-reported surveys for empathy level detection may not expose the real empathetic conduct of the scholars. The baseline status of the student’s mental illness was unknown due to ethical concerns. Last, our results on stress may be temporary, as PSS is used to quantify the feelings of anxiety associated with the previous month.

## CONCLUSION

In this study, the empathy scores increased from the start of undergraduate medical education and decreased significantly in the subsequent clinical years. Third-year students were found to be more stressed than in other academic years. The study showed there was no significant correlation between empathy and stress levels. Policymakers should take steps to modify stress management and promote empathetic clinical practice before entering the clinical years. The medical curriculum across Pakistan needs to be reformed, including empathy and professionalism, so that the professional quality of our future doctors is in line with international standards and values.

### Authors Contribution:

**SR:** Conceived & designed study, analysis & interpretation of data, responsible and accountable for the accuracy and integrity of the work

**MA:** Revising manuscript critically, responsible and accountable for the accuracy and integrity of the work.

**RN:** Reviewed final draft.

**AJ:** Data analysis & interpretation of data.
